# On the Relief of Excessive and Dangerous Tympanites by Puncture of the Abdomen

**Published:** 1888-12

**Authors:** 


*On the Relief of Excessive and Dangerous Tympanites by Puncture of the Abdomen.* By JOHN W. OGLE, M.D. Pp. 110. London: J. & A. CHURCHILL. 1888.

In a very learned treatise, embellished with classic quotations and a little scholastic philosophy, Dr. Ogle discusses the value of puncture of the abdomen for the relief of dangerous tympanites. The case is stated very fairly and honestly, for and against; the verdict of the author being favourable to the practice. In our view, even if we confined our attention to the evidence adduced, the verdict should have gone the other way.

But the whole question should have been narrowed down to more definite issues. Thus the author makes no attempt to separate distension by gas inside the bowels, from distension by gas or fluids lying free in the cavity; and he quotes cases of each variety as if they told equally in favour of tapping for tympanites. Then, again, he quotes various authors who have, chiefly in past times, spoken in favour of tapping strangulated hernia as an aid to reduction; and surely he ought to have added that at the present day the practice has been entirely discontinued. Comparisons with animals are fallacious: the anatomy is different, and the disease is not the same. These differences should have been mentioned in speaking of tapping in gaseous abdominal distension in horses, cattle, and sheep. Finally, some attempt should have been made to classify the cases suitable for the operation—if there are any such; and also to meet some of the objections which so many men have urged against the proceeding—such as, that a good few cases have died of it, from extravasation of fæculent fluids into the cavity; that, in addition to this positive danger to life, the relief in most cases is only temporary; and that it does not attack the origin of the disease, while it displaces other proceedings which might do so.

We would resort to the proceeding only in two classes of cases. The first in dying patients, who are suffering greatly from abdominal distension; here it is a mode of euthanasia: the second in cases of sudden and inexplicable distension, where life is endangered from syncope or asphyxia; here it is simply the speediest mode of averting death, while we wait for the opportunity when proper remedial measures may be adopted.

